# Sequence variability of *Campylobacter *temperate bacteriophages

**DOI:** 10.1186/1471-2180-8-49

**Published:** 2008-03-20

**Authors:** Clifford G Clark, Lai-King Ng

**Affiliations:** 1 Enteric Disease Program, National Microbiology Laboratory, Public Health Agency of Canada, 1015 Arlington St., Winnipeg, MB, R3E 3R2, Canada

## Abstract

**Background:**

Prophages integrated within the chromosomes of *Campylobacter jejuni *isolates have been demonstrated very recently. Prior work with *Campylobacter *temperate bacteriophages, as well as evidence from prophages in other enteric bacteria, suggests these prophages might have a role in the biology and virulence of the organism. However, very little is known about the genetic variability of *Campylobacter *prophages which, if present, could lead to differential phenotypes in isolates carrying the phages versus those that do not. As a first step in the characterization of *C. jejuni *prophages, we investigated the distribution of prophage DNA within a *C. jejuni *population assessed the DNA and protein sequence variability within a subset of the putative prophages found.

**Results:**

Southern blotting of *C. jejuni *DNA using probes from genes within the three putative prophages of the *C. jejuni *sequenced strain RM 1221 demonstrated the presence of at least one prophage gene in a large proportion (27/35) of isolates tested. Of these, 15 were positive for 5 or more of the 7 *Campylobacter *Mu-like phage 1 (CMLP 1, also designated *Campylobacter jejuni *integrated element 1, or CJIE 1) genes tested. Twelve of these putative prophages were chosen for further analysis. DNA sequencing of a 9,000 to 11,000 nucleotide region of each prophage demonstrated a close homology with CMLP 1 in both gene order and nucleotide sequence. Structural and sequence variability, including short insertions, deletions, and allele replacements, were found within the prophage genomes, some of which would alter the protein products of the ORFs involved. No insertions of novel genes were detected within the sequenced regions. The 12 prophages and RM 1221 had a % G+C very similar to *C. jejuni *sequenced strains, as well as promoter regions characteristic of *C. jejuni*. None of the putative prophages were successfully induced and propagated, so it is not known if they were functional or if they represented remnant prophage DNA in the bacterial chromosomes.

**Conclusion:**

These putative prophages form a family of phages with conserved sequences, and appear to be adapted to *Campylobacter*. There was evidence for recombination among groups of prophages, suggesting that the prophages had a mosaic structure. In many of these properties, the Mu-like CMLP 1 homologs characterized in this study resemble temperate bacteriophages of enteric bacteria that are responsible for contributions to virulence and host adaptation.

## Background

Though *Campylobacter *spp., especially *C. jejuni*, have been recognized as the most frequent cause of bacterial enteric infection in many countries [1-3], there is a great deal yet to learn about the ecology and pathogenesis of these organisms. Several *Campylobacter *genomes have now been fully or partially sequenced [4,5] and a number of microarray experiments have explored the genetic variability within the genus [6-8]. However, to identify novel genes within *Campylobacter *isolates of interest it will be necessary to either sequence more genomes or explore the roles of mobile genetic elements such as transposons, plasmids, and bacteriophages.

Lysogenic, or temperate, bacteriophages were first recovered from *Campylobacter fetus *(at the time known as *Vibrio fetus*) in 1968 after induction with mitomycin C, induction in aging cultures, or induction using co-cultivation methods [9]. Transduction of streptomycin resistance by phage induced with UV light was demonstrated shortly thereafter [10], indicating that *Campylobacter *temperate bacteriophages are capable of horizontal DNA transfer. Using co-cultivation techniques, Bryner and colleagues [11] induced, isolated, and characterized temperate bacteriophages from 22 of 38 strains of *Vibrio fetus *(*Campylobacter fetus*). Four groups of bacteriophage from lysogenic strains were defined on the basis of differential lysis of a panel of test isolates [12], suggesting considerable heterogeneity in the temperate phage population. Early investigations into the role of *C. jejuni *in enteric disease of children demonstrated the presence of temperate bacteriophages that mediated resistance to typing phages and were capable of lysing a stock strain of *C. jejuni *[13]. These phages caused spontaneous plaque formation of the host bacterium. Spontaneous release of temperate bacteriophage was found to have a role in autoagglutination of *Campylobacter *isolates [14].

Autoagglutinated bacteria appeared to be "leaky", and phage tail-sheaths were associated with bacterial cells.

After this initial work there was a period in which temperate or lysogenic bacteriophages were not demonstrated in *Campylobacter *spp. Several investigators attempted unsuccessfully to isolate and propagate temperate bacteriophages from *C. jejuni *[15,16]. However, DNA sequences homologous to Mu and other bacteriophages were detected in the genome of *C. hyoilei *[17]. The very recent demonstration of three distinct bacteriophage integrated into the genome of chicken isolate RM 1221 suggests that such prophages may be common and important for the biology of *C. jejuni *[4]. At least one of these three *Campylobacter jejuni *integrated elements (CJIEs) [6] is a Mu-like phage inducible with mitomycin C designated either CJIE 1 or *Campylobacter *Mu-like phage 1 (CMLP 1). Elements similar to these CJIEs were found quite frequently when a large panel of isolates was tested using a DNA microarray, and CMLP 1 appeared to integrate essentially randomly in the genome [6]. Results from Southern blotting using CMLP 1-homolog genes as probes also showed that this phage appears to be capable of loss and insertion or re-insertion into different parts of the *C. jejuni *genome, producing changes in pulsed-field gel electrophoresis (PFGE) patterns [18]. Genome rearrangements in *C. jejuni *were found to result from inter-genomic inversions between Mu-like prophages; activation of dormant Mu-like prophages subsequent to predation by virulent bacteriophage was also noted [19]. It appears that the prophages of at least some isolates were therefore functional and capable of lytic growth.

At the time this study was begun, there were no data available on the distribution or variability within *C. jejuni *of prophages homologous to CJIE 1, 2, and 4 of RM 1221. One of the questions we wanted to answer was whether these *Campylobacter *Mu-like prophages were similar in their heterogeneity to lambdoid prophages found, for instance, in many of the Enterobacteraceae. In this study we have therefore investigated the sequence diversity in approximately 9 – 11 kb regions of twelve bacteriophages homologous to CMLP 1 of *C. jejuni *strain RM 1221. Sequence variability due to apparent insertions and deletions was detected, and results supported the concept that these bacteriophages are modular in nature, with mosaic genomes. There was no evidence for the presence of lambdoid prophages similar to those found in the Enterobacteriaceae.

## Results

### Distribution of *Campylobacter *CJIE ORFs

**Figure 1:**
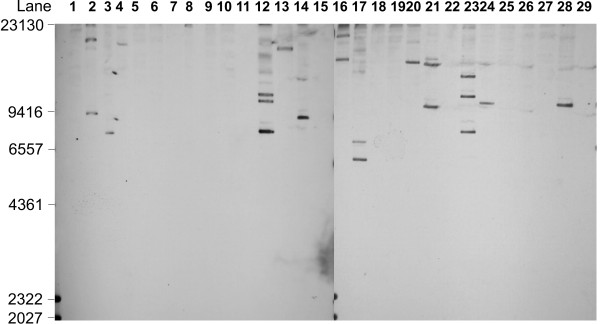
Southern blot showing hybridization of the probe for cje0215 of sequenced strain RM 1221 to DNA from isolates used in this study. Pst I cut genomic DNA was hybridized and blotted as summarized in the Methods section. Sizes of the Hind III-cut λ DNA standard are shown at the left of the figure. Lanes with visible bands were scored as positive for the presence of DNA sequence homologous to the probe, while those without visible bands were scored negative. Isolates were in lane: 1, NC 13254; 2, NC 13255; 3, NC 13256; 4, NC13257; 5, NC 13258; 6, NC 13259; 7, NC 13260; 8, NC 13261; 9, NC 13262; 10, NC 13263; 11, no DNA; 12, NC 13265; 13, NC 13266; 14 RM 1221; 15, no DNA; 16, 99–7046; 17, 00–0949; 18, 00–1597; 19, 00–2533; 20, 00–2538; 21, 00–2575; 22, 00–2814; 23, 00–2818; 24, 00–3477; 25, 00–4221; 26, 00–6200; 27, 03–1120; 28, RM 1221; 29, NC 13264.

ORFs for the three CJIEs were distributed differentially through the *Campylobacter *population tested (Figure 1, Table 1). CMLP 1 homologs were detected for all genes tested in 9 isolates, and the corresponding prophages were presumed to be intact, though not necessarily functional. Southern blots of 13 isolates showed no CMLP 1 gene probe hybridization, suggesting the prophages were completely absent. One to six of the CMLP 1 genes tested were missing in the remaining 13 isolates; some of these may be mosaic prophages while others, especially those with hybridization to only one or two probes, could be homologous genes from a source other than a CMLP 1-like prophage.

**Table 1:** Results of Southern blotting experiments using probes prepared from *C. jejuni *RM 1221. Shown are the RM 1221 prophages and the genes within each that were used to develop probes for Southern blotting. A "+" indicates that the gene was probe-positive in the isolate, while a "-" indicates the absence of reactivity with the probe.

				**CJIE1/CMLP 1**							**CJIE 2**		**CJIE 4**			**λ**
				
**Isolate No.**	**Location**	**Source**	**HS Serotype**	**cje0215**	**cje0221**	**cje0226**	**cje0232**	**cje0244**	**cje0251**	**cje0270**	**cje0544**	**cje0569**	**cje1418**	**cje1454**	**cje1471**	
RM 1221	NA	chicken	53	+	+	+	+	+	+	+	+	+	+	+	+	-
00–2818	Ontario	bovine	35	+	+	+	+	+	+	+	+	+	-	-	-	-
00–2425	Ontario	human	2	+	+	+	+	+	+	+	-	+	+	+	+	-
00–2538	Ontario	human	2	+	+	+	+	+	+	+	-	+	+	+	+	-
00–2544	Ontario	human	2	+	+	+	+	+	+	+	-	+	+	+	+	-
00–3477	Ontario	human	23,36	+	+	+	+	+	+	+	-	-	-	-	-	-
00–5700	Ontario	bovine	2	+	+	+	+	+	+	+	-	-	+	+	+	-
NC 13255	ND	human	19	+	+	+	+	+	+	+	-	-	-	-	-	-
NC 13256	ND	human	23	+	+	+	+	+	+	+	-	-	-	-	-	-
NC 13265	ND	human	53	+	+	+	+	+	+	+	+	+	-	-	-	-
00–0949	Québec	human	2	+	+	+	+	+	-	+	-	+	+	+	+	-
00–2575	Ontario	human	47 (*C. coli*)	+	+	+	+	+	-	+	-	-	-	-	-	-
00–6470	New Brunswick	human	2	+	+	+	+	+	-	+	-	+	+	+	+	-
99–7046	Louisiana	chicken	1	+	+	+	-	+	-	+	-	-	-	-	-	-
01–1512	New Brunswick	human	2	+	+	+	-	+	-	+	-	+	+	+	+	-
NC 13266	ND	human	41	+	+	+	-	+	-	+	-	-	-	-	-	-
00–2859	Ontario	bovine	2	-	+	+	+	+	-	-	-	+	+	+	+	-
00–3925	New Brunswick	human	2	-	+	+	-	-	-	-	-	+	+	+	+	-
NC 13257	MD	human	57	+	-	-	-	-	-	-	-	-	-	-	-	-
NC 13261	ND	bovine	50	-	-	-	+	-	-	-	-	-	-	-	-	-
NC 13262	ND	sand	NT	-	-	-	+	-	-	-	-	-	-	-	-	-
NC 13260	ND	ovine	5	-	-	-	-	-	-	+	-	-	-	-	-	-
NC 13264	ND	human	11	-	-	-	-	-	-	+	-	-	-	-	-	-
00–1597	Alberta	human	9,37	-	-	-	-	-	-	-	-	+	+	+	+	-
00–2426	Ontario	human	2	-	-	-	-	-	-	-	-	-	+	+	+	-
00–2533	Ontario	human	2	-	-	-	-	-	-	-	-	+	+	+	+	-
00–2814	Ontario	bovine	11	-	-	-	-	-	-	-	-	-	-	-	-	-
00–4221	Alberta	human	3	-	-	-	-	-	-	-	-	-	-	-	-	-
00–6200	Ontario	human	4,13	-	-	-	-	-	-	-	-	+	+	+	+	-
01–3648	Egypt	human	2	-	-	-	-	-	-	-	-	-	+	+	+	-
01–5949	Ontario	canine	2	-	-	-	-	-	-	-	-	-	-	-	-	-
03–1120	France	human	31	-	-	-	-	-	-	-	-	-	-	-	-	-
NC 13254	ND	bovine	50	-	-	-	-	-	-	-	-	-	-	-	-	-
NC 13258	ND	ovine	50	-	-	-	-	-	-	-	-	-	-	-	-	-
NC 13259	ND	human	18	-	-	-	-	-	-	-	-	-	-	-	-	-
NC 13263	ND	human	NT	-	-	-	-	-	-	-	-	-	-	-	-	-
81–176	NA	human	NA	-	-	-	-	-	-	-	-	-	-	-	-	-
NCTC 11168	NA	human	2	-	-	-	-	-	-	-	-	-	-	-	-	-

ND = not determined; NT = not typeable; NA = results not available

There did not appear to be a strong association of CMLP 1 carriage with the source, the HS serotype, or the phylogenetic background of the isolate. CJIE 4 was found somewhat less frequently, as it was detected in 14/35 isolates. One of the two CJIE 2 genes tested, cje0569, was also found in 14 isolates; however, the second ORF (cje0544) was detected in only 3 isolates. No sequences with homology to λ phage were found in any of the isolates tested in Southern blotting experiments.

All CMLP 1 gene homologs used for Southern blot experiments were found in two isolates from bovine stools, but were completely absent from other bovine isolates. The single isolate from a chicken that carried a CMLP 1 homolog was either missing some of the genes found in the RM 1221 prophage or had genes that were divergent in at least two regions of its sequence. Both ovine isolates did not appear to carry the phage, though one had a single phage gene homolog.

### Sequence analysis of CMLP 1 homologs

On the basis of the results of the Southern blot experiments described above, 12 isolates were chosen for sequence analysis of putative prophages homologous to CMLP 1. Our intent was to determine whether there was any sequence variability among these prophages and characterize the nature and extent of the changes present. If warranted, a subset of the prophages would then be completely sequenced at a later date. Since virulence genes are often inserted among the late genes of bacteriophage λ [20-23], we decided to sequence the region of the prophage containing genes for the prophage tail components, tail tape measure protein and virion morphogenesis protein.

Approximately 10 – 11 kb (~30%) of each prophage, equivalent to a region from about cje0215 to cje0232 of the RM 1221 genome, was sequenced by PCR amplification of approximately 1 – 3 kb fragments of the integrated phage genomes followed by chromosomal walking to fill in the complete sequence. In the case of isolate NC 13266 an amplicon was not obtained for the region adjacent to the cje0220 homolog, so that the sequenced region is shorter than those of the other CMLP 1 homologs. Different primer sets were used to obtain PCR amplicons from this region for the two main groups of isolates. Primer sets phs5 and phs6 proved capable of amplifying DNA from the group containing NC 13255, NC 13265, and other isolates, while the pcc1 primer set was used to amplify product from members of the group containing the Walkerton outbreak strain 1 isolates. The % G+C of the sequenced regions of the 12 prophages and the homologous region of RM 1221 CMLP 1 ranged from 30.66 to 31.93.

**Figure 2:**
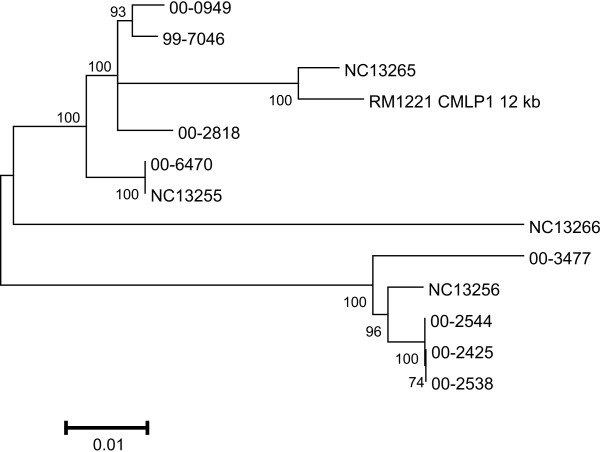
Dendrogram showing the phylogenetic relationships of the CMLP 1 homologs characterized in this study. The dendrogram was produced in MEGA 3.1 with untrimmed phage sequences, including that of NC 13266, by using the Neighbor-joining method with Kimura-2 parameter. The robustness of branches was tested by bootstrapping 1000 times.

The phylogenetic relationships among the CMLP 1 homologs are shown in Figure 2. The figure shows the relationships of the untrimmed sequences of all 12 prophages; a figure using all sequences (except that of NC 13266) trimmed to the same length was indistinguishable except for the lack of NC 13266 (data not shown). Three separate groupings were detected. One group contained the sequences of CMLP 1 homologs from the three Walkerton outbreak strain 1 isolates 00–2425, 00–2538, and 00–2544, which were indistinguishable over the entire length of the sequence tested. One of the MLST reference isolates, NC 13256, was also included in this group, as was isolate 00–3477. These latter two isolates had quite different STs, flaA SVR sequence types, and HS serotypes from the other isolates in this group. A second group of related prophage sequences included all other isolates except NC 13266, which constituted a group of its own.

**Figure 3:**
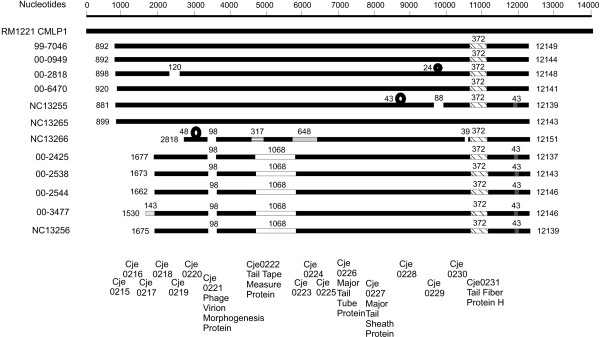
Schematic diagram of the major structural features of the 12 CMLP 1 homologs compared with the sequence of CMLP 1 from RM 1221. All isolates were numbered against the RM 1221 sequence beginning with the putative target site duplication starting at position 207005 in the RM 1221 genome, which becomes nucleotide 1 in the figure. The common backbone is shown in black, deletions by gaps in the sequence, insertions by loops above the backbone, and regions of divergent sequence are shown with different shading or patterns. Numbers above gaps, insertions, and divergent sequence indicate the length of the feature, while numbers on each side of the sequence show the position in the CMLP 1 sequence where each sequence begins and ends. Approximate locations of the phage coding regions are shown at the bottom of the figure.

The major structural features of the CMLP 1 homologs from all 12 isolates are compared with CMLP 1 in Figure 3. Isolate NC 13265 showed the strongest homology with CMLP 1 over its entire length. All other isolates had a 372 nucleotide region of divergent sequence in homologs to cje0231, which encodes the phage tail fiber protein H. The % G+C of the 372 bp insert was 37.94, much higher than the % G+C of the sequenced part of these prophages or of the *Campylobacter *chromosome as a whole. Isolates 00–2425, 00–2538, and 00–2544 had identical sequences in this insert. Other isolates had slightly less identity for this 372 bp insert: 98% in 00–3477, NC 13256, and NC 13266; 97% identity in 99–7046, 00–6470, and NC13255; and 96% identity in isolates 00–0949 and 00–2828. There were no other sequences closely related to this insert sequence found in BLAST (blastn) searches of the nr/nt database. The next most closely related oligonucleotide was a 58 bp sequence from the *Mus musculis *chromosome (accession number gb/AC163020.9/) that had 88% identity with the *Campylobacter *sequence.

The group of sequences that included the three Walkerton outbreak strain 1 isolates had a common 1068 nucleotide region of divergent sequence within cje0222 (tail tape measure protein). The % G+C of the insertion in isolate 00–2425 was 34.93, higher than the 31.66 % G+C of the entire sequenced part of this putative prophage. This insertion had an identical sequence over its entire length in isolates 00–2525, 00–2544, 00–2538, and NC 13256, and was 99% identical over its entire length with isolate 00–3477. These isolates were the only ones that had such a high degree of homology in BLAST (blastn) searches of the nr/nt database; the next most closely related sequence was a 58 nt fragment from a 2043 bp phage-related tail gene of a *Wolbachia *endosymbiont of *Drosophila melanogaster *(accession number gb/AF420275.1/AF420275).

Isolate NC 13266 had a different region of divergent sequence (648 nucleotides) with a % G+C of 25.89 that incorporated one end of cje0222 and adjacent genes. While the first 335 bp of this showed moderate homology with the homologous region in the other prophages, including RM 1221, the final 310 bp had no homology to any sequences in the nr/nt database and had a 22.58% G+C. Finally, all sequences in the group containing Walkerton outbreak strain 1 isolates, plus NC 13255, had a common divergent sequence of 43 nucleotides after the ORF homologous to cje0231. This short sequence had no homology to the consensus sequence of the other strains and only low homology to any other sequences in the nr/nt database.

Insertions of different sizes and at different locations were detected in NC 13266 (48 nucleotides), in NC13255 (43 nucleotides in cje0228), and 00–2818 (24 nucleotides near cje0229). Deletions were also found. A common deletion of 98 nucleotides was found in CMLP 1 homologs from 00–2425, 00–2538, 00–2544, 00–3477, NC 13255 and NC 13266. Isolate 00–2818 had a 120 nucleotide deletion within cje0220 and NC 13255 had an 88 nucleotide deletion near cje0229. In addition to the 98 nucleotide deletion described above, the CMLP 1 homolog of isolate NC 13266 had a 39 nucleotide deletion at the end of cje0230. It should be noted that, in addition to the insertions and deletions, all CMLP 1 homolog sequences contained single nucleotide changes not apparent on the schematic diagram shown in Figure 3.

Consistent with these major structural features, split decomposition analysis of the 12 partial prophage genomes exhibited a reticulate structure suggestive of extensive recombination among this population (data not shown).

### Presence of promoter elements upstream of open reading frames (ORFs) homologous to ORFs from isolate RM 1221

CMLP 1 from RM 1221 appeared to produce functional lytic phage particles [4] suggesting that the genes required for both the lysogenic and lytic life cycle were functional. However, we were unable to successfully induce and propagate any of the putative prophages in our isolates; these prophages may or may not be functional, and may represent remnant phage DNA present in the bacterial chromosome. Since there appeared to be a fair bit of DNA sequence diversity within our CMLP 1 homologs, we were interested in determining whether the predicted ORFs within the partial sequences of these prophage homologs included known *Campylobacter *promoter elements.

Most of the ORFs encoding genes with putative bacteriophage functions were preceded by the consensus ribosome binding sequence (AAGGA) for *C. jejuni *[24] in all isolates. Prophage ORFs with this sequence at the appropriate position in the promoter included cje0231, cje230 (except in isolate NC 13265), cje0228, cje0227, cje0226, cje0219, cje0217, and cje0216.

For the ORFs encoding the putative cje0220 homolog the situation was a little more complicated. In isolates 99–7046, 00–0949, 00–2818, 00–6470, NC 132355, and NC 13265 this ORF had four possible alternative start sites with the start codons TTG (beginning with amino acids LHSKEWSG...), GTG (beginning with amino acids VAFSDAT...), and ATG (providing the start for two sequence variants, one beginning with the amino acids MARKTKA and the other encoding the sequence homologous to that of the RM 1221 ORF beginning with MKNNT...). Only the GTG and latter ATG start codons were preceded by the consensus ribosome binding sequence. In the cje0220 homolog of isolates 00–2425, 00–2538, 00–2544, 00–3477, NC13256, and NC 13266 there were two predicted start sites; the first producing a peptide beginning with "MARK..." and the second producing the RM 1221-like peptide beginning with "MKNNT...". Both start sites were preceded by appropriate ribosome binding sequences, though their placement relative to the start codon differed.

For some other ORFs the consensus ribosome binding sequence was not present adjacent to the putative start codon, and possible ribosome binding sites containing a suitable combination of "A"s and "G"s – preferably containing a GG dinucleotide – were sought. The cje0229 homolog in each putative prophage was preceded by a sequence consisting of GGGAGAG, while each cje0225 homolog was preceded by GGGCA, cje0224 by AGAGG, cje0222 by AAAGGG in all isolates except NC 13266, and cje0221 by ATGGA. Only the promoter regions of cje0218 homologs were devoid of a sequence resembling the consensus ribosome binding site or one of the potential ribosome binding sites proposed here.

The -10, -16, and -35 regions of *Campylobacter *promoters are quite heterogeneous in both sequence and placement [24]. For this reason, only sequences identical to one of these 12 experimentally determined -10 sequences were sought within 100 nucleotides of the (putative) start codon(s). cje0231 homologs in all isolates were preceded by the *C. jejuni sodB *-10 sequence, TAATATT. A 2A12, *proA*-like -10 sequence was found preceding cje0226, cje0222, and only the ATG start codon encoding the "MKNNT..." start site of cje0220. The other two potential start sites for this ORF were not preceded by any of the known -10 promoter sequences.

cje0230 from all isolates except NC 13265 carried the orf1-like -10 promoter sequence, TATCTTT. Isolate NC 13265 had three different -10 sequences in its promoter region; these were identical to the sequences determined for *sodB *(TAATATT), *ileS *(TAGAATT), and *glyA *(TATTGTT). All cje0225 ORFs were preceded by the *lysS *-10 oligonucleotide (TTTAAAC), while cje0221 was preceded by the 23ES-like sequence (TACAATT) and cje0217 by the 1G9-like sequence (TATATTA). A large proportion of the ORFs identified may therefore be expressed though, once again, there is no evidence yet that these putative prophages are capable of being induced. ORFs corresponding to cje0229, cje0228, cje0227, cje0224, cje0219, cje0218, and cje0216 did not have any of the experimentally determined -10 promoter sequences upstream of the start codon. It is possible that many of these ORFs carry alternative -10 sequences.

### Variability of protein sequences and evidence for a modular structure of CMLP 1 homologs

**Figure 4:**
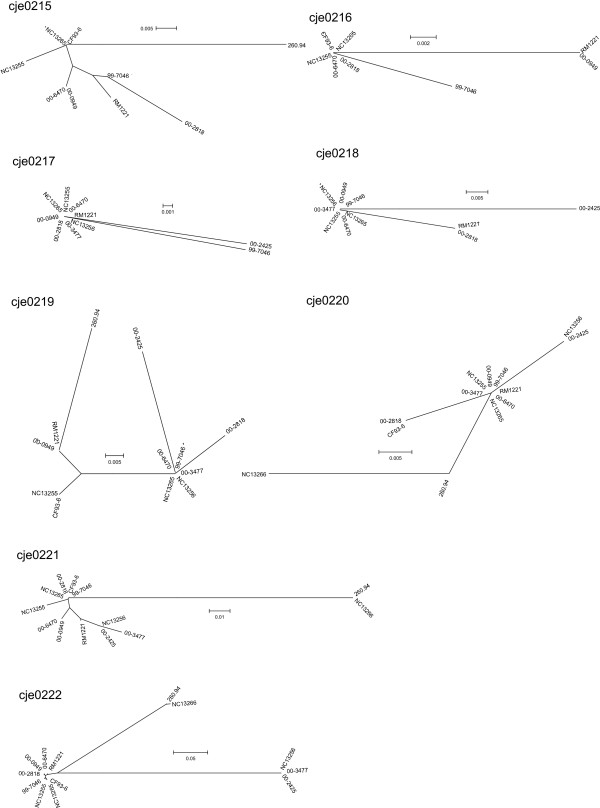
Phylogenetic relationships of proteins from CMLP 1 homologs. Dendrograms were produced in MEGA 3.1 using the Neighbor-joining method with Kimura-2 parameter. ORFs homologous to cje0215 to cje0222 of strain RM1221.

Translations were obtained using DNA sequences for each ORF corresponding to a CMLP 1 gene, and the resulting peptides were compared. Results of phylogenetic analysis of these proteins are shown in Figure 4. Translation products from CMLP 1 in isolate RM 1221 were included in the analysis, as were proteins from isolates 260.94 and CF93-6 that were identified in BLAST searches using the translated peptides from each CMLP 1 homolog, in each of the 12 isolates, as query sequences.

Most of the available prophage sequence was occupied with open reading frames corresponding to the CMLP 1 genes identified previously [4]. As noted in the previous section many, but not all, of the proteins homologous to those from CMLP 1 had readily identified consensus ribosome binding sequences [24]; fewer had clearly identifiable -10 consensus sequences. Full-length proteins were present in all cases but one (see below), indicating that there were no major errors in sequencing that resulted in abnormally truncated proteins. It should be noted that, because of the lack of variation in DNA sequences, the protein sequences of CMLP 1 homologs from isolates 00–2338 and 00–2544 are represented here by the sequence from 00–2425, to which they were identical.

Differences noted in the DNA sequence were apparent as differences in the translated protein products. The largest possible open reading frame present in cje0216 of isolate 00–2818 was 28 amino acids longer at the N-terminus than any of the cje0216 proteins from other isolates.

Interesting variations were found in cje0220, which produces a protein with homology to a DNA adenine methylase. The DNA insertion within this gene in NC 13266 produced a region of peptide homology with cje0220 of strain 260.94, which was quite different from all other cje0220 peptides. Only a partial protein sequence was obtained for the cje0220 homolog in NC 13266. The 120-nucleotide deletion within cje0220 of 00–2818 resulted in an in-frame deletion within the C-terminal third of the protein. Four possible start sites were evident in cje0220 proteins from isolates 00–0949, 00–2818, 00–3477, NC 132356, and NC 13265, especially when the alternative start codons (UUG/TTG and GUG/GTG), known to be functional in both *C. jejuni *and in Mu phages [24,25], were used. The first site would produce proteins beginning with a leucine, while the second would produce proteins beginning at the same site as in strain CF93-6, but beginning with a valine. The third putative start site would produce proteins beginning with a methionine (MARK...) that were 20 amino acids shorter than the first site, while the fourth start site would result in proteins beginning with a methionine at the same start site as cje0220 in RM 1221, an additional 26 amino acids shorter than proteins beginning at the second putative start site. Isolates other than those noted above had only the third and fourth putative start sites. It is not clear whether all these putative start sites are actually functional, but they do suggest the potential for additional variability based on fine sequence variation.

Variability was also evident in the protein sequences of cje0222, which was homologous to phage tail tape measure proteins. The C-terminal third of the protein was highly conserved in all sequences. In isolate NC 13266 the remaining sequence was very similar to that of strain 260.94 (see also Figure 4), consistent with the DNA sequence divergence noted previously and shown in Figure 3. The DNA sequence divergence identified in the group of isolates related to the Walkerton outbreak strain 1 isolates was apparent as a region of 336 amino acids that was conserved among these isolates and different from proteins in other strains. Interestingly, this 336 amino acid region had frequent, regular, periodic stretches of sequence identity with all the other proteins, perhaps necessary to maintain the overall structure and conserve the function of the protein.

Most peptide sequences of cje0228 were very similar, except that isolates 00–2425 (plus 00–2538 and 00–2544), 00–3477, NC 13255, and strain RM 1221 replaced the consensus C terminal peptide of KYKKM with NTKVKK. Interestingly, the 43 nucleotide insertion into cje0228 of NC 13255 translated into a C-terminal peptide extension of QKIQKIQKIQKIQKIQKM, a change one might expect would have some effect on protein function. Cje0229 of NC 13255 also had a C-terminal extension different from all the other proteins, which were quite conserved overall. The cje0230 peptide sequence was conserved except for the 13 amino acid deletion in NC 13266 corresponding to the 39 nucleotide in-frame deletion in the DNA of this isolate. The C-terminal third of cje0231 from RM 1221 and NC 13265 was different from the corresponding protein in all other isolates, which were very similar.

Homologs of proteins cje0217, cje0218, cje0219, cje0223, cje0224, cje0226, and cje0227 were remarkably well conserved overall, suggesting that the complete peptide sequence was required for the proper function of at least some of these proteins.

**Figure 5:**
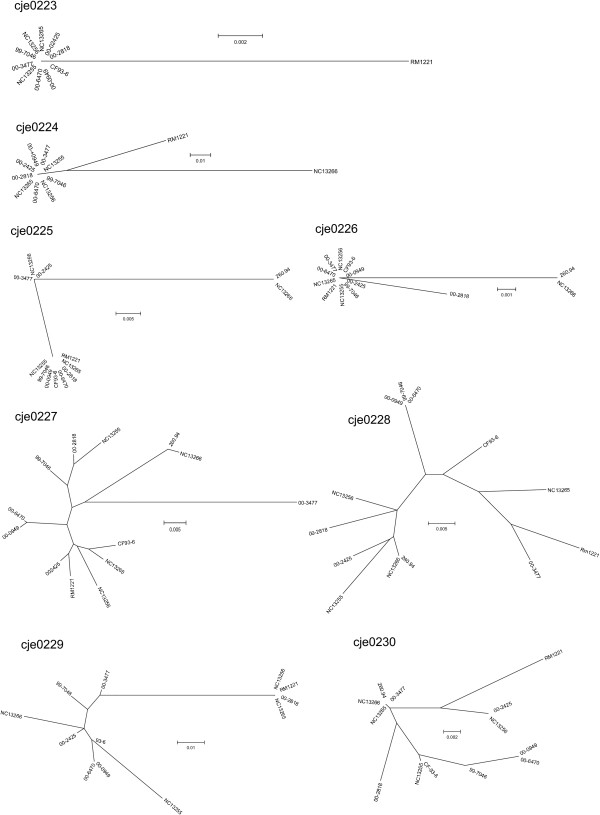
Phylogenetic relationships of proteins from CMLP 1 homologs. Dendrograms were produced in MEGA 3.1 using the Neighbor-joining method with Kimura-2 parameter. ORFs homologous to cje0223 to cje0230 of strain RM1221.

**Figure 6:**
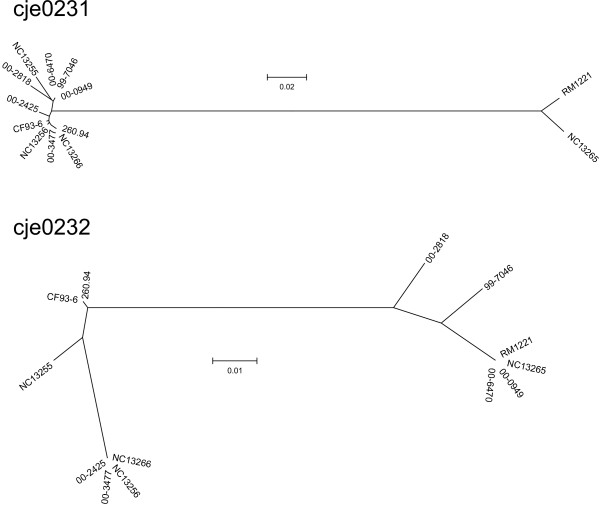
Phylogenetic relationships of proteins from CMLP 1 homologs. Dendrograms were produced in MEGA 3.1 using the Neighbor-joining method with Kimura-2 parameter. ORFs homologous to cje0231 and cje0232 of strain RM1221.

Phylogenetic comparisons for each protein (Figures 4, 5, 6) suggest that the CMLP 1-homologous prophages are mosaics of proteins/genes from different sources. Further evidence for the modularity of the CMLP 1 ORF homologs characterized in this study was demonstrated by performing BLASTP searches with each protein from each CMLP 1 homolog. The closest match was determined by the smallest E value. The phages appear to be mosaics, with different putative proteins similar to those from RM 1221, strain 93-6, and strain 260.94 (Table 2).

**Table 2:** Identification of known proteins with the closest homology for each protein from CMLP 1 homologs identified in this study.

	CMLP1 gene																	
Isolate	cje0215	cje0216	cje0217	cje0218	cje0219	cje0220	cje0221	cje0222	cje0223	cje0224	cje0225	cje0226	cje0227	cje0228	cje0229	cje0230	cje0231	cje0232
99–7046	RM 1221	93-6	RM 1221	RM 1221	93-6	RM 1221	93-6	93-6	RM 1221	RM 1221	RM 1221	RM 1221	RM 1221	93-6	93-6	93-6	93-6	RM 1221
00–0949	93-6	RM 1221	RM 1221	RM 1221	RM 1221	RM 1221	93-6	RM 1221	RM 1221	RM 1221	RM 1221	RM 1221	RM 1221	93-6	93-6	93-6	93-6	RM 1221
00–2818	RM 1221	93-6	RM 1221	RM 1221	93-6	93-6	93-6	RM 1221	RM 1221	RM 1221	RM 1221	RM 1221	93-6	260.94	RM 1221	260.94	93-6	RM 1221
00–6470	93-6	93-6	RM 1221	RM 1221	93-6	RM 1221	93-6	RM 1221	RM 1221	RM	RM 1221	RM 1221	RM 1221	93-6	93-6	93-6	93-6	RM 1221
NC 13255	93-6	93-6	RM 1221	RM 1221	93-6	RM 1221	93-6	93-6	RM 1221	RM 1221	RM 1221	RM 1221	93-6	260.94	RM 1221	93-6	93-6	93-6
NC 13265	93-6	93-6	RM 1221	RM 1221	93-6	RM 1221	93-6	93-6	RM 1221	RM 1221	RM 1221	RM 1221	93-6	93-6	93-6	RM 1221	RM 1221	RM 1221
NC 13266	ND	ND	ND	ND	ND	260.94	260.94	260.94	not found	RM 1221	260.94	260.94	260.94	260.94	93-6	260.94	260.94	260.94
NC 13256	ND	ND	RM 1221	RM 1221	93-6	RM 1221	RM 1221	RM 1221	RM 1221	RM 1221	RM 1221	RM 1221	93-6	260.94	RM 1221	260.94	260.94	260.94
00–3477	ND	ND	RM 1221	RM 1221	93-6	RM 1221	RM 1221	RM 1221	RM 1221	RM 1221	RM 1221	RM 1221	93-6	RM 1221	93-6	260.94	260.94	260.94
00–2425	ND	ND	RM 1221	RM 1221	93-6	RM 1221	RM 1221	RM 1221	RM 1221	RM 1221	RM 1221	RM 1221	RM 1221	260.94	93-6	260.94	260.94	260.94

It should be noted that a number of additional potential ORFs producing translation products between 3,000 and 10,000 Daltons were detected, especially when alternative start codons were included in the analyses. For the most part these overlapped the previously characterized proteins on the opposite strand and lacked clearly identifiable ribosome binding sites, and so are not discussed in detail here. Examples include two ORFs that were found in most or all prophage sequences, encoded in the 00–2425 prophage by nucleotides 1680 to1979 and by nucleotides 2487 to 2729. If expressed, these ORFs would produce proteins with molecular masses of 7,653 and 8,369, respectively, that have no homology with any known peptide sequences. In contrast, an ORF encoded by nucleotides 2928 to 3167 of isolate 00–2425 appeared to be present only in 00–2425, 00–2538, 00–2544, 00–3477, and NC 13256. This ORF, if expressed and functional, could contribute to differences in biology among the two groups of isolates.

## Discussion

The finding of CJIEs 1, 2, and 4 in a number of *Campylobacter *isolates confirms the observation of Parker et al. [6] that these elements appear to be quite widespread in the population. These elements also appeared to be present in similar proportions of the population in the present work and that of Parker et al. [6].

Prophages homologous to CMLP 1 from strain RM 1221 were found in 4/6 isolates of Walkerton outbreak strain 1, suggesting that the rate of loss or gain of this bacteriophage may be quite high, as previously suggested [18]. The fact that two of the Walkerton outbreak strain 1 isolates lacked prophage could mean that the prophage had no effect on virulence of the isolates. However, it is not know whether the prophage was lost from these isolates before infecting humans or upon culture and subculture in the laboratory; no firm conclusions on the association of the presence of the prophage and the virulence of isolates can be drawn. The prophage was also not found in the single human isolate of Walkerton outbreak strain 2 (00–2533) tested, and was not present in a number of other isolates obtained from human stools. However, too few animal isolates were tested to draw any conclusions about the association of CMLP 1 and its homologs with particular hosts.

It was interesting that no lysogenic phage DNA homologous to phage λ was found in the genome of any isolate, as this phage family plays the predominant role in the pathogenesis of many members of the Enterobacteraceae. *C. jejuni *and *C. coli *share environmental niches, including the human intestinal environment, with *Salmonella *and *E. coli *isolates carrying lambdoid prophages partially responsible for the virulence of these organisms. The complete absence of lambdoid prophages from *Campylobacter*, if proven true in the long term, could indicate that these bacteria lack receptors for infection by lambdoid phages, that there are effective barriers to lysogeny by these phages, or that the genes carried by these phages do not provide a sufficient selective advantage to the organism to be stably maintained in the population.

If one assumes that the presence of genes from the beginning, middle, and end of CJIE 4 indicates the presence of the whole element, it can be seen that this element is present in a number of isolates from which CMLP 1 (CJIE 1) is absent. This in turn indicates that these elements can be inherited independently, though the data do not allow a distinction between the existence of CJIE 4 as a mobile element versus the differential carriage of the two elements through gain or loss of CJIE 1. Results from Southern blots using probes to CJIE 2 are somewhat more difficult to interpret due to the fact that the two genes tested are present in different frequencies in the population. Future work will concentrate on this element alone.

Phages homologous to CMLP 1 were found in 4 of 13 reference isolates from the UK that represent much of the variability within *C. jejuni *found by MLST [26]. The isolates carrying these genes were from different geographic sources than either strain RM 1221 or the isolates analyzed in this work, suggesting that variants of this bacteriophage are widely distributed in *C. jejuni *populations and that the phage may have been acquired early in the evolution of this organism. It was considered somewhat surprising, therefore, that only three major groups of CMLP 1 homologs were found when DNA sequences were analyzed phylogenetically. Sequence comparisons indicated that the CMLP 1 homolog carried by isolate NC 13266 showed evidence of recombination with the other two groups of phage as well as the acquisition and incorporation of novel DNA sequences. This was supported by our inability to obtain sequence further upstream of the cje0220 gene with primers that produced PCR amplicons from other isolates, a finding that suggests that this region contains unique DNA sequences in NC 13266. The NC 13266 CMLP 1 homolog appeared to be highly related to strain 260.94 in all proteins for which sequence was available from GenBank. Future work will involve cloning and sequencing further regions of NC 13266 using strain 260.94 sequences for developing PCR primers.

CMLP 1 homologs from isolates 00–2425, 00–2538, 00–2544, 00–3477, and NC 13256 had similar structural features, including a 98 bp deletion and a 1068 bp sequence quite distinct from the analogous sequence in CMLP 1. This 1068 bp sequence, as well as a 372 bp sequence replacement common to most prophages analyzed in this study, had a somewhat higher % G+C than the overall prophage sequence and the *C. jejuni *genome as a whole, suggesting that these sequences were acquired from an organism other than *C. jejuni*. Recombination appeared to have a major role in the diversification of the prophages under study. Because the 372 bp replacement sequence was found in all prophages except NC 13265 and RM 1221, we would speculate that this event may have occurred first in the evolution of the prophages studied here. The 43 bp replacement was present in only a subset of these prophages, and would appear to have been acquired later. Finally, the 1068 bp replacement was found in only a small subgroup containing the three Walkerton outbreak isolates, suggesting that it was acquired last. Most of the insertions and deletions appear to be strain-specific, and suggest fairly frequent changes in the prophage DNA sequence. The finding of the 98 bp deletion in both NC 13266 (in the absence of the 43 bp replacement) and in the group containing the Walkerton isolates (which all contained the 43 bp replacement) suggests that at least some of the prophage sequence changes could be modified by additional recombination events. This makes the construction of a tidy evolutionary tree difficult for these integrated bacteriophages.

The 100% sequence identity of isolates 00–2425, 00–2538, and 00–2544 supports the classification of these isolates as clones of the Walkerton outbreak strain 1 [18,27]. The other Canadian isolate from this group, 00–3477, was recovered in Ontario but, in typing studies, had different genetic and phenotypic characteristics from the Walkerton outbreak strain 1 isolates. The final isolate (NC 13256) with a DNA sequence similar to this group was recovered in the UK in 1991, and so represents an isolate from quite a different geographic location and time. It seems as though the different prophage families characterized in this work have become geographically widespread. Though the sampling of putative prophages included here is very small and only part of the phage genome was sequenced in each case, it is reasonable to conclude that there is a global distribution of *Campylobacter *temperate phages with similar sequences. It is also possible that there are more families of sequence variants yet to be discovered.

It was interesting that the start codons preceding ORFs in most prophage genes, including those of CMLP 1 in RM 1221, were identical to consensus start codons for *Campylobacter*. Since CMLP 1 was previously induced from RM 1221 [4], this observation suggests that at least some of the prophage homologs found in other isolates may also be competent for induction and the subsequent production of infectious phages. It also suggests that these Mu-like prophages are well adapted to *Campylobacter *and that *Campylobacter *may be the preferred or unique host of these phages, an hypothesis supported by the fact that the % G + C of the sequenced part of the prophages is very close to that of previously sequenced *C. jejuni *genomes (30.6% G+C for NCTC11168 [5] and 30.31% for RM 1221 [4]). At this time, however, we have no experimental evidence to support this possibility, and the prophage DNA in these isolates may be part of inactive prophage remnants. Induction of prophages and infection of *Campylobacter *spp. and other genera with the resulting infectious bacteriophage particles will be the subject of future work.

The effect of variations in phage DNA sequence on the resulting protein sequence was readily apparent. Some proteins had amino acid sequences quite different from the corresponding RM 1221 sequence, so much so that one would expect the function of the protein to be highly modified or abrogated. Most insertions and deletions were in-frame, so that a full-length protein was made. In these cases, it is possible that the expressed proteins might have modified function. Several proteins appeared to have potential N-terminal extensions compared with the RM 1221 homolog, though it is not clear whether these would be included in the final protein product. Alternate start codons were responsible for the possible presence of some of these N-terminal peptide extensions. These data confirm observations previously submitted by others to GenBank for isolate CF93-6. Further proteomics and functional studies will be necessary to answer some of the questions raised by the sequence analysis presented here and elsewhere.

Bacteriophages have critically important roles in genome diversification and the evolution of virulence and host adaptation of other enteric bacteria [20,23,28,29]. Genes encoding Shiga toxins (Stx) 1 and 2 are found on lambdoid phages in Shiga-toxigenic *Escherichia coli*, while similar Gifsy and Fels phages encode a number of virulence factors in *Salmonella enterica *serovar Typhimurium [21]. Some of these prophage-encoded *S*. Typhimurium proteins are effectors translocated into eucaryotic cells by type three secretion systems encoded at other locations in the chromosome [30]. In addition to carrying genes encoding virulence factors, integrated prophage can affect gene expression of the host bacterium [31,32]. While much less is known about the role(s) of Mu phages than lambdoid phages in enteric bacteria, it is possible that Mu phages also affect the biology and virulence of their host in similar ways. This work shows that the putative *Campylobacter *prophages exhibit at least some of the properties, including a modular or mosaic structure, in common with prophages from other enteric bacteria, thereby supporting the possibility that they may also have similar functions in virulence and the biology of the bacteria. No morons that could be novel virulence genes were found during the partial sequencing of the CMLP 1 prophage homologs in *C. jejuni *isolates. To address whether additional novel genes might be present in other parts of the prophage, future work will involve completion of the DNA sequences of a subset of the prophages discussed here. It would also be of interest to determine, using expression DNA microarrays, quantitative RT-PCR, and 2D-DIGE, whether prophage genes are expressed and whether the presence of integrated Mu-like prophages affects the expression of *Campylobacter *chromosomal genes. Initial work can be done using a naturally occurring strain pair with and without the prophage [18], though ideally isogenic strains would be created by phage transduction of a prophage-negative isolate. The fact that these prophages appear to be fairly common suggests that they confer biological properties that can be advantageous to the bacterial host under some circumstances. Determining what those circumstances are may provide valuable insight into the biology and virulence of human-pathogenic *Campylobacter*.

## Conclusion

CMLP 1 and its homologs appear to represent a temperate bacteriophage family that is widely distributed and frequently carried within the *Campylobacter jejuni *population. Phages in this family appear to have undergone some differentiation, and may be continuing to evolve. Future studies are required to understand how these bacteriophages interact with their host bacteria, and what implications this has for the pathogenesis, ecology, survival, and growth of these bacteria.

## Methods

### Isolates and culture conditions

Table 3 contains a list of isolates used for these studies. *C. jejuni *and *C. coli *isolates were chosen from among isolates previously characterized as part of investigations into a large Canadian water-borne outbreak [27]. *C. jejuni *MLST reference isolates representing most of the major clonal complexes of this organism [26] were purchased from the National Collection of Type Cultures (NCTC; London, U.K.) and are designated with "NC" before the isolate number in Table 3. K. Rahn at the Laboratory for Foodborne Zoonoses, Guelph, Ontario, Canada provided the genome sequenced-strain RM 1221 [4]. All isolates were stored in glycerol peptone water (25% v/v glycerol, 10 g/L neopeptone, 5 g/L NaCl) at -80°C. Cultures were grown on Mueller-Hinton agar (Oxoid Inc., Nepean, Ontario, Canada) containing 10% sheep erythrocytes at either 37°C or 42°C in a microaerobic atmosphere (10% CO_2_, 5% O_2_, and 85% N_2_).

**Table 3:** Isolates used in this study.

Isolate	Species	Source	Location	Biotype	ST^§^	flaSVR type	HS serotype	HL	PFGE	PFGE	Walkerton
								serotype	*Sma *I	*Kpn *I	outbreak strain
99–7046	*C. jejuni*	chicken	Louisiana	II	925	36	1	NT	ND	ND	NA
00–0949	*C. jejuni*	human	Québec	ND	8	356	2	36	9	32	NA
00–1597	*C. jejuni*	human	Alberta	I	930	9	9,37	NT	ND	ND	NA
00–2425	*C. jejuni*	human	Ontario	II	21	36	2	125	1	1	1
00–2426	*C. jejuni*	human	Ontario	II	21	36	2	125	2	2	1
00–2533	*C. jejuni*	human	Ontario	hipp. neg.	169	41	2	4	3	3	2
00–2538	*C. jejuni*	human	Ontario	II	21	36	2	125	11	1	1
00–2544	*C. jejuni*	human	Ontario	II	21	36	2	125	4	1	1
00–2575	*C. coli*	human	Ontario	I	new 2	357	47	34	10	4	NA
00–2814	*C. jejuni*	bovine	Ontario	II	928	16	11	82	ND	ND	NA
00–2818	*C. jejuni*	bovine	Ontario	II	933	122	35	51	21	ND	NA
00–2859	*C. jejuni*	bovine	Ontario	II	21	36	2	112,125	4	1	1
00–3477	*C. jejuni*	human	Ontario	II	new 1	274	23,36	5	ND	ND	NA
00–3925	*C. jejuni*	human	New Brunswick	ND	21	356	2	100	26	31	NA
00–4221	*C. jejuni*	human	Alberta	I	931	222	3	94	ND	ND	NA
00–5700	*C. jejuni*	bovine	Ontario	II	21	36	2	1	4	1	1
00–6200	*C. jejuni*	human	Ontario	2	806	41	4,13	7	30	ND	NA
00–6470	*C. jejuni*	human	New Brunswick	ND	8	356	2	36	9	32	NA
01–1512	*C. jejuni*	human	New Brunswick	II	8	356	2	90	26	31	NA
01–3648	*C. jejuni*	human	Egypt	I	21	53	2	128	49	37	NA
01–5949	*C. jejuni*	canine	Ontario	II	21	49	2	128	50	38	NA
03–1120	*C. jejuni*	human	France	II	474	34	31	NT	ND	ND	NA
NC 13254	*C. jejuni*	bovine	ND	ND	21	140	50	ND	ND	ND	NA
NC 13255	*C. jejuni*	human	ND	ND	22	232	19	ND	ND	ND	NA
NC 13256	*C. jejuni*	human	ND	ND	42	239	23	ND	ND	ND	NA
NC 13257	*C. jejuni*	human	ND	ND	45	70	57	ND	ND	ND	NA
NC 13258	*C. jejuni*	ovine	ND	ND	48	32	50	ND	ND	ND	NA
NC 13259	*C. jejuni*	human	ND	ND	49	11	18	ND	ND	ND	NA
NC 13260	*C. jejuni*	ovine	ND	ND	52	57	5	ND	ND	ND	NA
NC 13261	*C. jejuni*	bovine	ND	ND	61	42	50	ND	ND	ND	NA
NC 13262	*C. jejuni*	sand	ND	ND	177	77	NT	ND	ND	ND	NA
NC 13263	*C. jejuni*	human	ND	ND	206	58	NT	ND	ND	ND	NA
NC 13264	*C. jejuni*	human	ND	ND	257	16	11	ND	ND	ND	NA
NC 13265	*C. jejuni*	human	ND	ND	354	100	53	ND	ND	ND	NA
NC 13266	*C. jejuni*	human	ND	ND	362	338	41	ND	ND	ND	NA

^§ ^HS refers to heat stable serotyping of the lipooligosaccharide antigen, HL refers to heat labile (Lior) serotyping, ST refers to the sequence type obtained by DNA sequencing using the Oxford multi-locus sequence typing (MLST) method, flaA SVR refers to the results from sequencing of the flagellar (*flaA*) short variable region (SVR), and PFGE is pulsed-field gel electrophoresis. "NT" means not typeable, "ND" means not determined, and "NA" means not applicable.

### PCR and DNA sequencing

Template DNA was prepared from bacteria using the PureGene™ Genomic DNA purification kit (Gentra Systems, Minneapolis, MN) according to the manufacturer's recommendations. DNA from strain RM 1221 was used as a positive control in all reactions and strains NCTC 11168 and 81–176 were used as negative controls. A control in which water was substituted for DNA template was included in all runs. 100 μl PCR reaction mixtures consisted of 1× PCR buffer, 2 mM MgCl_2_, 0.5 μM of each primer, 0.2 mM dNTP mix, 200 to 1000 ng of template DNA, and 5 U FastStart DNA polymerase (Roche, Laval, PQ, Canada). PCR was performed using a Perkin Elmer 2400 thermocycler. Denaturation and annealing times were 1 min each, while extension times were 1 min for amplicons less than 1 kb and 3 min for products larger than that. Products were electrophoresed on 1.5 % agarose gels then purified using the QIAquick PCR purification kit (Qiagen, Mississauga, ON, Canada) or Montage™ PCR Centrifugal Filter Devices (Fisher Scientific Inc., Edmonton, AB, Canada) according to the manufacturers' instructions. Sequencing reactions were run using Big Dye Terminator 3.1 Cycle Sequencing kits (Applied Biosystems, Streetsville, ON, Canada) according to the manufacturer's instructions, and sequencing was performed using an ABI 3100 or 3730 DNA Analyzer (Applied Biosystems).

Some investigators have had difficulties inducing *Campylobacter *prophages and keeping them in a form suitable for transducing recipient strains [15,16]. We therefore adopted a strategy of amplifying fragments of the prophage of approximately one to three kb and sequencing the products. The primers used to amplify the PCR products were also used for the initial sequencing reactions for that product; subsequent sequencing was based on walking the chromosome using primers made to newly sequenced regions of the phages. PCR primers and annealing conditions can be found in Table 4.

**Table 4:** Primer sequences and characteristics. Primers were used to generate PCR amplicons for uses as probes for Southern hybridizations or as template for DNA sequencing. Primers were based on the bacteriophage genes present in RM 1221 (Genbank accession number CP000025) and were selected with the aid of the PrimerSelect™ program in the Lasergene DNASTAR™ version 5.06 software. phig3r and phig4r primers were used with the phig2f primer to amplify regions that could not be amplified with the phig2f/phig2r combination.

Primer Name	Sequence 5' → 3'	Annealing temperature (°C)	Amplicon size	Position in RM1221 genome
cje0215F	GCG AGT GAA GGC AAA AG	47	351 bp	207652 – 207668
cje0215R	TTC TCC ATA GCA AGT GAT AAA C			208002 – 207981
cje0221F	ATT ATG GCG GGT GCT GGA G	45	330 bp	210639 – 210657
cje0221R	GAC TTT GTT ATT ATC TAT GG			210328 – 210347
cje0226F	TGG CGA AGT TAT ACA GGA AGG T	50	207 bp	214289 – 214310
cje0226R	AAG AAA GCC GCA TAA AGC ACT			214104 – 204124
cje0232F	TAA ACC ACC ATC CAA AAC AAA G	50	296 bp	219214 – 219235
cje0232R	TCC GCC ATA ATT AAA CCA CTC			218940 – 218960
cje0244F	TAC CGC TAT TTA TCC CCT GTG T	50	359 bp	223836 – 223857
cje0244R	ATT AGC GCC CAT TCT TTT TG			224175 – 224194
cje0251F	ATG GGG ATA AAT TTA GCA CTT G	50	392 bp	230266 – 230287
cje0251R	TAG GCC TTT AAC TTC ACT TTC AC			230657 – 230636
cje0270F	TTC ACC GCA AAG ATA AAA CTA A	50	373 bp	241603 – 241582
cje0270R	ACT ATA ATA TCA GCT GGG GAA CTA			241231 – 241254
cje0544F	AAT AGG GGA ATG CCA AAA A	45	357 bp	498853 – 498871
cje0544R	CTA CTA ATC TCA AAT ATC CTA CAT			499209 – 499186
cje0569F	ATT AAC TTC AGA TAT TTC CCA GAT	47	420 bp	513074 – 513097
cje0569R	AAC AGC CAT TTT TGA TAC TAC AG			512678 – 512700
cje1418F	ATC CGT TAC TTT CCT TAG CAG AGC	51	540 bp	1335107 – 1336130
cje1418R	CAT TAC CGG GGC GTT GTG			1336629 – 1336646
cje1454F	TAG TGG CTT ATC TTT AGT C	43	158 bp	1354180 – 1354198
cje1454R	ATT TCC TTG CTT TTA TT			1354041 – 1354057
cje1471F	CCG CAA ATG AAA CCG AAC AA	52	475 bp	1369607 – 1369626
cje1471R	GCC ATA ACC CAA AGC AGG ATT			1369151 – 1369171
phs5f	TGC TAG GCT TTG GGG TTT GTT	52	2679 bp	209776 – 209796
phs5r	AGC GCT GAA GAT GTG GGA GAT AG			212432 – 212454
phs6f	GTT TAG GCG GAG GCG GAA TA	51	2340 bp	207887 – 207906
phs6r	GTC GGG CGT GGT CTT TAG TGA			210206 – 210226
pcc1f	TAT GTG AAA TTA GTT GGC GAA GAT	51	3654 bp	208668 – 208691
pcc1r	TTG TGA GCA GAA TTG GAG GAA			in 00–2425
phs0f	GCC ATT CTT GCT AAC ACT TTT TGA	51	1559 bp	210155 – 210178
phs0r	AAT GGG GTT TTA GGA GGA CTT AT			211691 – 211713
phs1f	AAG GCT TTA AGG GAG GTT GTG T	52	2918 bp	211484 – 211505
phs1r	TCT GAT GAA TGG GCG AGT GA			214382 – 214401
phig2f	AGA AAG CCG CAT AAA GCA CT	52	1069 bp	214104 – 214124
phig2r	AAA AGC AAA GCA CAA AAC AGG			215153 – 215173
phig3r	AAA AGC CAA ACA TAA AAC AGG			
phig4r	AAA AGC CAA GCA TAA AAG AGG			
phig1f	GGC TTT AAA ACC CCG CTA CTA	51	814 bp	214161 – 214181
phig1r	TGG CCA CAG GTT CAA ATC TTA			214954 – 214974
phs2f	CCC ACC CCA AGA GCGATA AC	52	2783 bp	214711 – 214730
phs2r	AGG ATG AAG TGA GCG ATG AGC A			217472 – 217493
phs3f	AAC CCC CAT ATT GTC CAC	49	2863 bp	216133 – 216150
phs3r	CTT TAA GAG CCG TAT TTC CTA			218975 – 218995

### Southern blotting

Southern blotting was done using bacteria embedded in plugs essentially according to the methods for manual ribotyping of Clark et al. [33]. Bacterial cultures were grown for 48 at 37°C on Mueller-Hinton agar (Oxoid Inc., Nepean, Ontario, Canada) containing 10% sheep erythrocytes in a microaerobic atmosphere (10% CO_2_, 5% O_2_, and 85% N_2_). Bacteria were embedded in 1.2% Seakem Gold agarose (Mandel Scientific Co. Inc., Guelph, Ontario, Canada) plugs and these plugs were digested to lyse bacterial cells using the standardized CDC protocol [34]. Three-quarters of each original plug was equilibrated for 1 h with 1 × buffer H (Roche Molecular Biochemicals, Laval, Quebec, Canada). Digestion of DNA was accomplished by adding 40 U *Pst *I and 1.0 μl of a 0.5 mg/ml RNase solution (Roche Molecular Biochemicals, Laval, Quebec, Canada) in a total volume of 100 μl followed by an incubation period of 4 h at 37°C. Finally, the intact plugs containing digested DNA were equilibrated with 0.5 ml of 0.5X TBE (10 × TBE was obtained from Sigma-Aldrich Canada Ltd., Oakville, Ontario, Canada) for 15 min at room temperature and placed into a 1% agarose gel. Samples were electrophoresed for 18 h at 60 volts in 0.5 × TBE. Gels were stained with ethidium bromide, photographed, and blotted using a Vacugene XL blotting apparatus (Amersham Pharmacia Biotech Ltd., Baie d'Urfé, Quebec, Canada). Blotting was performed according to protocol No.1 of the manufacturer's instructions. Blots were cross-linked using UV and probed with 500 ng of labelled probe and 100 ng of labelled λ DNA.

Probes were generated by PCR amplification of bacteriophage genes from either *C. jejuni *strains RM 1221 or isolate 00–2544 using the primers and conditions listed in Table 4. Primers were designed based on the bacteriophage genes present in RM 1221 (GenBank accession number NC_003912), amplified by PCR, and purified as summarized above. Probes (amplification products) were sent to the DNA Core Facility at the National Microbiology Laboratory for DNA sequence analysis to confirm the identity of the product prior to use in hybridizations. Probe preparation, hybridization and detection were performed using the AlkPhos direct labelling and CDP-*Star *detection system (Amersham, GE Healthcare, Baie D'Urfe, PQ, Canada) under low stringency conditions according to the manufacturer's instructions. Strains RM 1221, NCTC 11168, and 81–176 were included on blots as positive and negative controls; RM 1221 was included on each blot analyzed, while NCTC 11168 and 81–176 were present only on one set of blots. The size standard used was DNA Molecular Weight Marker II (bacteriophage lambda [λ] DNA cut with Hind III; Roche). All blots were also developed using only λ DNA as a probe. This provided a test for the presence of λ prophages and further served to ensure that no bands due to potentially lysogenic lambda phages were seen when blots were developed with probes for RM 1221 phage genes.

### Analysis of DNA and protein sequences

Sequence assembly and editing were done using Seqman™ and EditSeq™ in the Lasergene DNASTAR™ version 5.06 software (DNASTAR Inc., Madison, Wis). The sequences obtained had at least two strands in opposite directions, while some regions had four or five overlapping contigs.

Sequences were saved as FASTA (.fas) files. After entry into MEGA™ 3.1 [35] the sequences were aligned using Clustal W and dendrograms were constructed using the Neighbor-Joining method with Kimura 2 parameter. The robustness of branches was tested with bootstrapping using 1000 simulations. Protein sequences were similarly entered into the software and analyzed. Blast searches of database nr were carried out through the NCBI website [36] and E values were used to determine the known peptide most closely matching the protein(s) of interest. Split decomposition analysis was performed using the program Splitstree™ 4.0 using only parsimony informative sites.

Prophages were searched for the presence of known ribosome binding sites and -10 promoter sequences [21]. Additional sequences were tentatively identified as possible ribosome binding sites if they: 1) contained a mixture of A and G residues; 2) were found no further than 10 nucleotides upstream of the putative start codon of each open reading frame (ORF); and 3) were conserved in a majority of promoters for each ORF. Each of the individual -10 promoter regions identified previously by Wösten et al. [24] was considered to be present and potentially active if it was located no further than 100 nucleotides upstream of the putative start site. Several open reading frames had more than one potential start codon, especially when the alternate start codons UUG/TTG and GUG/GTG known to be used by *Campylobacter *[24] and Mu [25] were included in the analysis.

### Accession numbers

Sequences have been deposited in GenBank and are numbered EF694684 – EF694695.

## Authors' contributions

CGC was responsible for conception of the study, experimental design, data collection, and analysis. L-KN participated in data analysis and preparation of the manuscript.
